# Determinants of person-centered maternity care at the selected health facilities of Dessie town, Northeastern, Ethiopia: community-based cross-sectional study

**DOI:** 10.1186/s12884-020-03221-2

**Published:** 2020-09-10

**Authors:** Fentaw Teshome Dagnaw, Sofonyas Abebaw Tiruneh, Melkalem Mamuye Azanaw, Aragaw Tesfaw Desale, Melaku Tadege Engdaw

**Affiliations:** Department of Public Health, College of Health Sciences, Debre Tabor University, Debre Tabor, Ethiopia

**Keywords:** Person Centered Maternity Care, Mother, Ethiopia

## Abstract

**Background:**

Person-centered maternity care is providing care that is respectful and responsive to individual women’s preferences, needs, and values and ensuring that their values guide all clinical decisions during childbirth. Although person-centered health care is one of the factors that increase client satisfaction and increased health service utilization in Ethiopia, little is known about predictors of person-centered maternity care. Therefore, the aim of this study was to identify the determinant factors of person-centered maternity care among mothers who gave birth in selected health facilities in Dessie town, Northeastern, Ethiopia.

**Methods:**

A community-based cross-sectional study was conducted with a total of 317 study participants at Dessie town selected by a simple random sampling technique. The data was coded and entered Epi-data version 4.4 and analyzed using SPSS version 23. Descriptive statistics was presented using tables and figures. Multivariable linear regression analysis was used to identify factors associated with Person-Centered Maternity Care. Two sides P-value < 0.05 was taken to declare statistically significant.

**Results:**

Overall, 310 study participants participated with a response rate of 97.8%. In multivariable linear regression, rural residence (β = -4.12; 95% CI: -7.60, -0.67), family average monthly income ≤ 3000 birr (β = -6.20, 95% CI: -9.40, -3.04), night time delivery(β = -2.98, 95%CI: -5.90, -0.06), dead fetus outcome during delivery (β = -12.7; 95% CI: -21.80, -3.50), and 2–7 days health facility length of stay (β = -5.07, 95% CI: -9.20, -0.92) were significantly decreased Person Center Maternity Care score, whereas private health institution delivery (β = 14.13, 95% CI: 7.70, 20.60) is significantly increased Person centered maternity care score.

**Conclusions:**

This study revealed that most of the factors that affect person-centered maternity care are modifiable factors. Therefore, Primary attention should be given to improve the quality of care through effective communication between clients and providers at each level of the health care delivery system to increase the uptake of high-quality facility-based births.

## Background

According to the Institute of Medicine (IOM), Person Centered Maternity Care (PCMC) is defined as providing maternity care that is respectful and responsive to individual women’s preferences, needs and values; ensuring that their values guide all clinical decisions before, during and after childbirth [1]. A study in low and middle-income countries showed that women across all settings were not receiving person-centered maternity care. In addition, Communication between mothers and health care providers, respecting women’s autonomy and provision of dignified and supportive care were tend to be poor across those countries [2]. Evidence from East and South Africa indicated that many women did not have an explanation on the labor process, did not hear about the findings of their examinations and did not ask if they had any questions, with a prevalence of 42% in Rwanda and 16% in Ethiopia [3].

Previous studies showed that 66% and 57% of the mothers experienced PCMC at Addis Ababa and Bahir Dar cities respectively [4, 5]. Other study conducted in Ethiopia showed that person-centered health care is one of the factors that increase client satisfaction and affect the health-seeking behavior of the community [6]. Disrespect and physical abuse were common barriers to utilization of maternal health services and affects the quality of care[7]. Moreover, women’s poor experiences with care at health institutions may be deterring them from seeking childbirth services and undermining existing national efforts to prevent maternal mortality in Ethiopia [8].

Facility-based childbirth and effective implementation of women’s rights increased by advancing PCMC approaches in maternal health services [4, 9]. In low and middle-income countries, PCMC was higher among wealthier, employed, literate, and married women [2]. A community survey in Tanzania revealed that women with secondary education were more likely to report abusive treatment [10]. The study conducted in Kenya showed that significant differences exist in PCMC scores by socio-demographic characteristics like:- women who are married, college-educated, literate, wealthier and hold salaried jobs or have a trade reported, on average, higher PCMC than women who are unmarried, less educated, illiterate, poorer and working in agriculture respectively [11]. The study done in Bahir Dar town showed that respondents who were from the rural area were more likely to report disrespect and abuse [5]. In LMIC, women received care from two providers of different genders reported a higher PCMC score than those who were assisted by only male or female providers [2]. According to WHO effective communication and continuity of care with sensitive, caring, kind, skilled, and competent staff have a great impact on a person-centered childbirth experience [12].

A study conducted in Ethiopia reported that women’s reporting of disrespect and abuse was significantly associated with childbirth complications, weekend delivery, no previous delivery at the facility, and experience of complications [8]. Respondents’ mode of delivery, time of delivery, complication during delivery and intention to give birth in a health institution were some of the factors associated with disrespect and abuse. Similarly, respondents who gave birth through caesarian section were more likely to experience disrespect and abuse than those respondents who gave birth through vaginal delivery [5].

Women who delivered in health community-based or private health facilities reported a higher PCMC score than those who delivered in public hospitals [2, 11]. Although several studies were conducted to identify determinants of PCMC in different countries, the determinants are varying from region to region depending on the socio-demographic and other factors. Moreover, evidence-based information on the determinants of person-centered maternity care is limited in the study area. Hence, we aimed to assess the determinants of PCMC among mothers who gave birth in health facilities of Dessie Town.

## Methods and materials

### Study design, area and period

A community-based cross-sectional study design was employed at Dessie town, Amhara regional state, Northeastern, Ethiopia. Dessie town is located 488 kilometers from Bahir Dar, the capital city of Amhara region, and 401 kilometers away from Addis Ababa. The town has 5 sub-cities and 26 kebeles (the smallest administrative division in Ethiopia); of which 18 kebeles are urban and eight kebeles are rural. According to Dessie Town administration health office, the total population of the town was 223,639 of them 112,938 were females). The numbers of women who were in the childbearing age group (15–49) were 52,734. There are 2 government hospitals, 8 public health centers, 3 private hospitals, and 45 private clinics in the town. The study was conducted from March-April 11/2019.

### Population, Sample Size determination and sampling procedure

The source populations were all mothers who gave birth at health institutions of Dessie town and randomly selected mothers who gave birth nine weeks prior to the data collection period were included as study population.

The sample size for this study was determined based on a single population proportions formula with assumption of the standard normal distribution corresponding to 95% confidence level, a margin of error assumed to be 5%, an assumption that the proportion of women reporting person-centered care while giving birth 57% was taken from the previous study conducted to assess the status of compassionate and respectful maternity care at Bahir Dar city in 2017[5]. Thus; considering non-response of 10%, the final sample size was 317.

The study participants were selected after the total number of mothers who gave birth nine weeks prior to the data collection period in health institutions of Dessie town at each kebeles was identified. The total number of mothers who gave birth in the town health facilities from January 01/2011-March 3/2011 E.C was 1,227 (i.e. nine weeks prior to the data collection period). Then, a simple random sampling technique was used to select individual study participants from the delivery registers of urban health extension workers of each kebeles, which contains the address (house number) of mothers.

### Data collection tool and procedures

The data were collected by face to face interview using semi-structured questionnaires composed of socio-demographic characteristics of the mother, obstetric history questionnaires and person-centered maternity care scale that was validated in Kenya and India to measure Person centered maternity care for developing settings. The scale has good internal consistency reliability, with Cronbach’s alpha above 0.80; and high content, construct, and criterion validity and includes 30 items that span three domains: dignity and respect (6 items), communication and autonomy (9 items), and supportive care (15 items). The tool was recommended to be administered to women who have recently given birth up to 9 weeks post-partum[13, 14].

Internal consistency/reliability for this study was checked by calculating Cronbach’s alpha for each of the domains to examine the extent to which respondents answered consistently to the theoretically similar items in each domain and it was found 0.81 for dignity and respect, 0.84 for communication and autonomy, 0.85 for supportive care sub-scales and 0.86 for full PCMC scale. The data collectors were four BSc nurses and two other BSc nurse supervisors were also assigned to follow the data collection process.

### Study variables

**Fig. 1:**
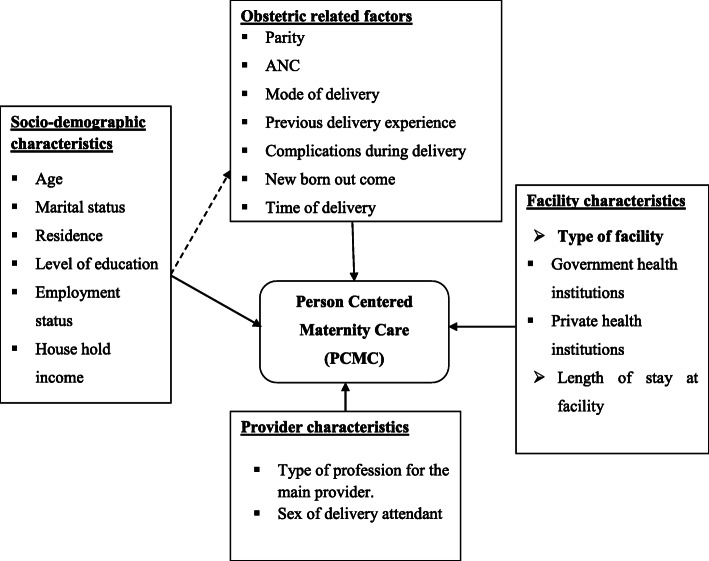
The conceptual framework for the predictors of Person Centered Maternity Care [4, 11–17].

The dependent variable of this study is Person centered maternity care and after reviewing different kinds of literature [4, 11–17] the independent variables were thematized into four categories (socio-demographic factors, obstetric related factors, facility-related factors, and provider-related factors) (Fig. 1). These factors include age, marital status, level of education, employment status, religion, residence, household average monthly income, parity, ANC, mode of delivery, previous deliveries experience, Complications during delivery, type of facility, length of stay in the facility, type of profession to the main provider and gender of the delivery attendant.

### Operational definition

#### Person-Centered Maternity Care

Was measured using PCMC scale which has three domains: dignity and respect, communication and autonomy, and supportive care and 30 items with each item have a four-point response scale. i.e. 0 (“no, never”), 1 (“yes, a few times”), 2 (“yes, most of the time”) and 3 (“yes, all the time”), and with negative items reverse coded (i.e. questions that were framed negatively, such as the physical, verbal abuse, auditory privacy and crowdedness of room questions, had to be recoded so that high numbers represent good care). Therefore, the scale score ranges from zero to 90.

#### Dignity and respect

Six items with each four-point response scale; i.e. 0 (“no, never”), 1 (“yes, a few times”), 2 (“yes, most of the time”) and 3 (“yes, all the time”) was used to measure dignity and respect. So, the total score ranges from 0 to 18.

#### Communication and Autonomy

Measured using nine items with each item has a four-point response scale; i.e. 0 (“no, never”), 1 (“yes, a few times”), 2 (“yes, most of the time”) and 3 (“yes, all the time”). So, the total score ranges from 0 to 27.

#### Supportive care

Measured using 15 items with each item has a four-point response scale; i.e. 0 (“no, never”), 1 (“yes, a few times”), 2 (“yes, most of the time”) and 3 (“yes, all the time”). So, the total score ranges from 0 to 45 [18].

### Data processing and Analysis

The collected data were entered into Epi-data version 4.4.1 and exported to SPSS version 23 software for analysis. Any errors identified during data entry were corrected by reviewing the original completed questionnaire. Descriptive statistics were used to describe the socio-demographic and reproductive characteristics of mothers. Simple and multiple linear regression analysis were fitted after creating dummy variables to identify the factors associated with the outcome variable. But, before fitting the linear regression model, the assumption of linearity was checked and satisfied using a scatter plot, normality was checked by plotting histogram and P-P plots and homoscedasticity was satisfied by plotting two scatter plots of standardized residuals against the standardized predicted values and it was randomly distributed.

The Durbin Watson statistic (acceptable range is 1.5 to 2.5) was used to check the assumption of independence of errors and autocorrelations. The value of the Durbin Watson statistic for this data was 1.6. Therefore, this analysis satisfied the assumption of independence and no autocorrelations. Multicollinearity assumption was checked through the Variance Inflation Factor (VIF) (acceptable range is less than 10). Hence, the maximum VIF score was for this research was 1.8, which showed no evidence for multicollinearity.

Then, factors that showed significant associations in a simple linear regression model were added to multiple linear regression models to control the potential confounding factors using the enter method. Variables that had a *P*-value of < 0.05 in the multivariable model were considered as statistically significant. Finally, results were compiled and presented using tables, graphs, and texts.

### Data quality management

The questionnaire was first prepared in English and then translated into Amharic and then back into English to ensure consistency by different experts for first and second translation. The questionnaire was pretested on 10% of the actual sample size (32 mothers) prior to the actual data collection period at Dessie town but they were not included in the actual data collection time and the correction was made accordingly. The training was given for both data collectors and supervisors for one day by the principal investigator. There was supervision daily and checking on 10% of the collected questionnaire. Finally, error reports were checked after entry to Epi-data using each case code for the quantitative data.

### Ethical considerations

Ethical clearance and approval to conduct this research were obtained from the Ethical Review Committee of Jimma University with reference number IHRPGS/221/2019. Permission to conduct the study also obtained from Dessie town health department. Verbal informed consent was obtained from the study participants in which all participants were aged above 16 years old and this was confirmed by the ethical committee. Confidentiality and anonymity were ensured throughout the execution of the study.

## Results

### Socio-demographic and obstetric characteristics of respondents

Three hundred ten mothers participated in this study with a response rate of 97.8%. Nearly three-fourths of the respondents 223 (71.9%) were from urban kebeles and the rest 87 (28.1%) were from rural kebeles of Dessie Town. The mean age of the respondents was 27.65 (SD ± 5.6) years with a minimum and maximum age of 17 and 48 years respectively. Most of the respondents were currently married 280 (90.3%). Most of the respondents 189 (61%) were Muslims and 104 (33.5%) were Orthodox followers. Regarding the level of education of mothers, almost half 154 (49.7%) of the respondents were secondary and above level. More than three-fourth 240 (77.4%) of mothers were not employed in formal governmental and non-governmental organizations. The mean monthly income was 3786 Ethiopian birr and more than half of the respondents (51.3%) income was ≤ 3000 Ethiopian birr (Table 1).

**Table 1:** Socio-demographic characteristics of respondents in Dessie town, Northeast Ethiopia, 2019 (*n* = 310)

Variables	Category	Frequency	Percentage (%)
Residence	Urban	223	71.9
	Rural	87	28.1
Age of mothers	16–19	15	4.8
	20–29	196	63.2
	≥ 30	99	31.9
Marital status	Currently married	280	90.3
	Currently unmarried	30	9.7
Mother’s religion	Muslim	189	61
	Orthodox Christian	104	33.5
	Protestant	12	3.9
	Catholic	5	1.6
Level of education	Unable to read and write	51	16.4
	Primary level (1–8 grades)	105	33.9
	Secondary and above	154	49.7
Employment status	Unemployed	240	77.4
	Employed	70	22.6
Income (in Ethiopian Birr)	≤ 3000	159	51.3
	> 3000	151	48.7
**Total**		**310**	**100**

From the total respondents, almost all 305 (98.4%) had a history of ANC follow up for recent most delivery. Besides, the majority of 199 (64.2%) mothers who received ANC service were seen at governmental health facilities. Over half of the respondents, 173 (55.8%) were Multiparous. Nearly a quarter of the respondents 69 (22.6%) had greater than four visits for ANC service. About two hundred fifty-five (82.3%) of a mother had the previous history of institutional delivery at least two children. Two hundred fifty-three (81%) of the mothers gave birth at governmental health institutions. Over half of the respondents, 159 (51.6%) and 162 (52.3%) of the delivery service was attended by a midwife and through spontaneous vaginal delivery respectively. A little over half 157 (50.6%) of the respondents reported that they gave birth at daytime and the majority 220 (71%) had no complications (Table 2).

**Table 2:** Obstetric characteristics of respondents in Dessie town, Northeast Ethiopia, 2019

Variables	Category	Frequency	Percentage (%)
Antenatal care	Yes	305	98.4
	No	5	1.6
Frequency of ANC	≤ 4	236	77.4
	> 4	69	22.6
Place of ANC	Government health institution	199	65.2
	Private health institution	106	34.8
Parity	Primiparous	137	44.2
	Multiparous	173	55.8
Total number of facility-based childbirth	≤ 2	255	82.3
	3–4	53	17.1
	> 4	2	0.6
Place of last delivery	Government health institutions	251	81
	Private health institutions	59	19
Profession of delivery attendant	Doctor	130	41.9
	Midwife	159	51.6
	Others (Nurse, HEW)	20	6.5
Sex of main provider	Male	186	60
	Female	102	32.6
	Both	23	7.4
Type of last delivery	Normal delivery	162	52.3
	Cesarean delivery	45	14.5
	Instrumental	103	33.2
Time of delivery	Day time	153	49.4
	Night time	157	50.6
Complication during delivery	Yes, for self	36	11.6
	Yes, for baby	35	11.3
	Yes, for both self and baby	19	6.1
	No	220	71
Newborn outcome	Alive	302	97.4
	Dead	8	2.6

### Person-Centered Maternity Care (PCMC) scales and sub-scales

The maximum and minimum score for PCMCs was 89 and 20 respectively (out of 90). The mean PCMC score of the respondents was 58 with SD = 13.9 from 90.

Standardization of the mean score was made by the following formula;

$$ \mathrm{Percentag}\kern0.17em \mathrm{means}\kern0.17em \mathrm{score}=\frac{\mathrm{Actual}\ \mathrm{score}\hbox{-} \mathrm{potential}\ \mathrm{minimum}\ \mathrm{score}}{\mathrm{Potential}\ \mathrm{maximum}\ \mathrm{score}\hbox{-} \mathrm{potential}\ \mathrm{minimum}\ \mathrm{score}}\;\mathrm{X}\;100\% $$

**Fig. 2:**
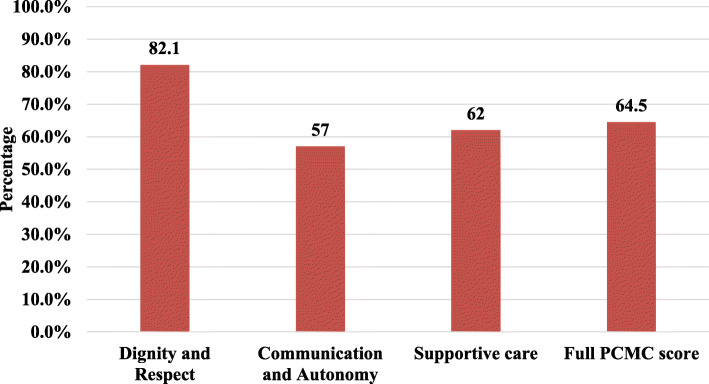
Distribution of percentage mean score of PCMC full scale and sub scales from the total expected score among mothers of Dessie town, 2019.

The Percentage mean score of PCMC scale of the respondents was 64.5% from the total expected score. Whereas, the percentage means score sub-scales were 82%, for dignity and respect, 57% for communication and autonomy, and 62% for supportive care (Fig. 2).

####  Dignity and respect

The mean score of the respondents was 14.79 (SD = 2.76). About one hundred ten (35.5%) of respondents in Dessie town felt they were treated with respect all the time and 84 (27.1%) of mothers reported they were treated in a friendly manner all the time during their stay in the health facilities. More than 56 (18.1%) and 21 (6.8%) of women reported that they were experienced verbal abuse and physical abuse at least once during their stay at the health institution respectively. Record confidentiality and auditory privacy in the in-depth interview were reported less frequently during labor and delivery (Table 3).

**Table 3:** Distribution of dignity and respect items (*n* = 310), Dessie Town, 2019

Items	No, never (%)	Yes, few times (%)	Yes, most of the time (%)	Yes, all the time (%)
Treatment with respect	4 (1.3)	53 (17.1)	153 (46.1)	110 (35.5)
Treatment in a friendly manner	11 (3.5)	75 (24.2)	140 (45.2)	84 (27.1)
Providers shouted, scolded, insulted, threatened, or talked rudely during treatment (Verbal abuse) (RC)	239 (77.1)	56 (18.1)^a^	15 (4.8)	0
Providers treated me roughly like pushed, beaten, slapped, pinched, and physically restrained (Physical abuse) (RC)	275 (88.7)	21 (6.8)^a^	14(4.5)	0
Feeling of other people not involved in care could hear the discussion with health care provider (Auditory privacy) (RC)	263 (84.8)	37 (11.9)	7(2.3)	3 (1)
Feel health information was/will be kept confidential (Record confidentiality)	10 (3.2)	10 (3.2)	165 (53.2)	125 (40.3)

^a^Shows that Yes, few times changed into Yes, once*RC* Reverse Coded

###  Communication and autonomy

The mean score of the respondents was 15.35 (SD = 5.25). Most respondents 200 (64.5%) reported that providers never introduce themselves when they first came to see them. Fifty-one 16% of mothers reported that providers never called them by their names. Only 46 (14.8%) of women in Dessie town felt that providers involved them in decisions about their care all the time whereas, 25 (8.1%) of them reported that they never involved in decisions about their care. About 22 (7.1%) of the respondents did not feel they could be in a position of their choice during delivery. Additionally, 46 (14.8%) and 38 (12.3%) of the respondents reported that health care providers never asked permission/consent during the examination and did not tell why they are doing such examinations/procedures respectively (Table 4).

**Table 4:** Distribution of communication and autonomy items, Dessie Town, 2019 (*n* = 310)

Items	No, never (%)	Yes, few times (%)	Yes, most of the time (%)	Yes, all the time (%)
Providers introduced themselves^a^	200 (64.5)	53 (17.1)	37 (11.9)	20 (6.5)
Providers called me by my name^a^	51 (16.5)	58 (18.7)	125 (40.3)	76 (24.5)
Feel involved in decisions about my care	25 (8.1)	77 (24.8)	162 (52.3)	46 (14.8)
Consent to examinations and procedures	46 (14.8)	80 (25.8)	116 (37.4)	68 (21.9)
Allowed position of choice	22 (7.1)	83 (26.8)	138 (44.5)	67(21.6)
Spoken in a language I understand	4 (1.3)	33 (10.6)	77 (24.8)	196 (63.2)
Examinations and procedures were explained	38 (12.3)	93 (30)	119 (38.4)	60 (19.4)
Purpose of medicines was explained#	27 (8.7)	97 (31.3)	136 (43.9)	44 (14.2)
Feel comfortable to ask questions I had to Providers	11(3.5)	69 (22.3)	126 (40.6)	104 (33.5)

^a^The choice of the item was changed into (No, none of them, Yes, few of them, Yes, most of them and Yes, all of them) #Did not get any medicine 6 (1.9%)

###  Supportive care

The mean score of the respondents was 27.87 (SD = 7.4). About 144 (46.5%) of women reported that they were not allowed to be with someone they wanted during labour and nearly 216 (69.7%) of women delivering without a companion. Besides, 94 (30.3%) of women reported that they felt that the rooms were crowded during their stay at the facility. Less than half 145 (46.8%) of the respondents reported that water was available all the time during their stay in the health institutions. Only 114 (36.8%) of the respondents reported that they thought there were enough health staff in the facility to care for them (Table 5).

**Table 5:** Distribution of supportive care items, Dessie Town, 2019 (*n* = 310)

Items	No, never (%)	Yes, few times (%)	Yes, most of the time (%)	Yes, all the time (%)
Allowed a labor companion^a^	144 (46.5)	49 (15.8)	56 (18.1)	55 (17.7)
Allowed a delivery companion	216 (69.7)	38 (12.3)	37 (11.9)	19 (6.1)
Providers talk to me about how I was feeling	20 (6.5)	62 (20)	160 (51.6)	68 (21.9)
Providers supported me when I had anxieties and fears	22 (7.1)	54 (17.4)	159 (51.3)	75 (24.2)
Feel providers did their best to control my pain	17 (5.5)	56 (18.1)	138 (44.5)	99 (31.9)
Providers paid attention when I needed help	11 (3.5)	60 (19.4)	130 (41.9)	109 (35.2)
Feel providers took the best care of me	11 (3.5)	52 (16.8)	148 (47.7)	99 (31.9)
Trust providers with regards to my care	4 (1.3)	54 (17.4)	135 (43.5)	117 (37.7)
Feel there were enough providers to care for me	10 (3.2)	67 (21.6)	119 (38.4)	114 (36.8)
Feel facility was crowded (RC)	94 (30.3)	65 (21)	99 (31.9)	52 (16.8)
Facility had water	33 (10.6)	58 (18.7)	74 (23.9)	145 (46.8)
Facility had electricity	2 (0.6)	25 (8.1)	48 (15.5)	235 (75.8)
Feel safe in the facility	11 (3.5)	54 (17.4)	156 (50.3)	89 (28.7)
Feeling about waiting time	Very long (%)	Somewhat long (%)	Little long (%)	Very short (%)
	39 (12.6)	91 (29.4)	109 (35.2)	71 (22.9)
Thinking the general environment of the health facility; the facility was	Very dirty (%)	Dirty (%)	Clean (%)	Very clean (%)
	11 (3.5)	54 (17.4)	203 (65.5)	42 (13.5)

^a^I did not want someone to stay with me 6 (1.9%)*RC *Reverse Coded

### Factors associated with Person-Centered Maternity Care

In simple linear regression analysis, residence of mothers, family average monthly income, level of education, ANC follow-up, place of ANC follow-up, Place of delivery, mode of delivery, sex of health care provider attending delivery, time of delivery, length of stay at the health institution and newborn outcome were significantly associated with PCMC score. In multivariable linear regression analysis, rural in residence, family average monthly income ≤ 3000 Ethiopian birrs, private health institution delivery, a night time of delivery, 2–7 days length of health institution stay, and dead delivery outcome in a health facility were significantly associated with PCMC score.

Keeping other variables constant, mothers living in the rural area had decreased the PCMC score by a factor of four times as compared to mothers living in an urban area (β = -4.16, 95% CI: -7.60, -0.67). Mothers having average monthly income ≤ 3000 Ethiopian birrs had decreased the score of PCMC by a factor six times as compared to mothers had greater than 3000 Ethiopian birrs average monthly income (β = -6.19, 95% CI: -9.40, -3.04). Mothers gave birth at private health institution were increase the PCMC score by a factor of 15 times as compared to their counterparts (β = 14, 95% CI: 7.70, 20.60). The person-centered maternity care score decreased by 3 times when mothers gave birth during the night time (β= -2.98, 95% CI: -5.90, -0.06). Keeping all other variables constant, mothers stay at the health institutions 2–7 days decrease the PCMC score by a factor of 5 times as compared to their counterparts (β = -5.07, 95% CI: -9.20, 0.92). Furthermore, mothers gave dead fetus decrease the PCMC score by a factor of 13 times as compared to mothers gave alive birth (β = -12.70, 95% CI -21.80, -3.50) (Table 6).

**Table 6:** Multivariable regression analysis factors for Person Centered Maternity Care scale, Northeast Ethiopia, 2019

Variables	Category	Unstandardized Adjusted β Coefficients	95% CI of β
	(Constant)	71.4	(67.70, 75.10) ***
Residence	Urban	0	
	Rural	-4.12	(-7.60, -0.67) *
Level of education	Secondary and above	0	
	Unable to read and write	-4.21	(-8.80, 0.40)
	primary Level (1–8 grades)	-2.64	(-6.10, 0.78)
Employment status	Non-employer	0	
	Employer	2.88	(-0.68, 6.44)
Income	> 3000 ETB	0	
	≤ 3000 ETB	-6.2	(-9.40, -3.04) ***
ANC follow-up	No	-4.38	(-16.30, 7.50)
	Yes	0	
Place of ANC	Government health institutions	0	
	Private health institutions	-2.07	(-7.90, 3.70)
Place of delivery	Government health institutions	0	
	Private health institutions	14.13	(7.70, 20.60) ***
Mode of delivery	SVD	0	
	Cesarean section	2.74	(-2.30, 7.80)
	Instrumental	-3.17	(-6.50, 0.15)
Time of delivery	Day	0	
	Night	-2.98	(-5.90, -0.06) *
Outcome of delivery	Alive	0	
	Dead	-12.7	(-21.80, -3.50) **
Sex of main delivery attendant	Male	0	
	Female	2.92	(-0.31, 6.14)
	Both	3.20	(-2.30, 8.80)
Length of stay at the health facility	≤ 1 day	0	
	2-7days	-5.07	(-9.20, -0.92) *
	Greater than one week	-5.58	(-13.30, 2.12)

NB: *CI *Confidence Interval, * = significant at *P*-value < 0.05, ** - significant at *P*-value < 0.01*** = significant at *P*-value < 0.001, *ETB *Ethiopian Birr

## Discussion

Our study investigated factors that can affect Person centered maternity care among mothers who gave birth in health institutions in Dessie town at the community level.

This study revealed that from rural kebeles and whose family average monthly income ≤ 3000 Ethiopian birrs had significantly lower person-centered maternity care score as compared with respondents from urban kebeles and whose family average monthly income > 3000 Ethiopian birr. This result is consistent with studies done in Ethiopia and Kenya in which lower monthly income participants have experienced more disrespect and abuse thane higher income participants [4, 5, 13]. This might be due to the reason that respondents from urban kebeles and with high monthly income had better confidence and experience or better tie to get services from health institutions through good communication with health care providers.

Additionally, respondents who gave birth at private health institutions had significantly higher person centered maternity care scores when compared with respondents who gave birth in governmental health institutions [4, 5, 13, 19]. This might be because private health institutions give more attention to person-centered care to attract more clients and for sustainable utilization of their services and the quality of services providers of private health institutions might be better than that of public providers.

Respondents who gave birth at night had significantly lower person-centered maternity care scores compared with respondents who gave birth at the daytime. This might be because the numbers of health professionals were small and overloaded with work at night time so, the providers might not give satisfactory services.

Respondents whose length of stay was 2–7 days had significantly lower person-centered maternity care scores as compared with respondents whose length of stay was less than or equal to one day. This is consistent with the study done in Bahir Dar and Tanzania [10, 17]. The reason for the similarly might be due to as the length of stay increases the probability of experiencing none-person-centered approach of care increases.

Respondents whose delivery outcome was dead had significantly lower person-centered maternity care scores compared with whose delivery outcome was alive. This is consistent with the one study done in Bahir Dar [5]. the reason might be due to mothers who lost their newborn might be dissatisfied with care given by the health professionals or they might think that they lost their newborn due to poor care they received within the health institutions.

## Conclusion and recommendations

Maternal place of residence, family average monthly income, time of delivery, place of delivery, length of stay at health institution and newborn outcome were factors that were significantly associated with person-centered maternity care. Overall, the result of this study showed that most modifiable factors affected person-centered maternity care in the area. Therefore, emphasis should be given to improve the quality of care through effective communication between clients and providers at each level of the health care delivery system to increase the uptake of high-quality institution-based births. Health care providers should also provide compassionate and respectful care for all mothers who give birth in health institutions regardless of their socio-economical background.

## Data Availability

The data is available from the corresponding author and will be provided upon a reasonablerequest.

## Abbreviations

ANC: Antenatal Care
BSc: Bachelor of Science
CI: Confidence Interval
D&A: Disrespect and Abuse
IOM: Institute of Medicine
KMS: Kilometers
LMICs: Low- and Middle-Income Countries
PCMC: Person Centered Maternity Care
RMC: Respectful Maternity Care
WHO: World Health Organization

## References

[1] Berwick DM (2009). What “patient-centered” should mean: Confessions of an extremist. Health Aff.

[2] Afulani PA, Phillips B, Aborigo RA, Moyer CA (2019). Person-centred maternity care in low-income and middle-income countries: analysis of data from Kenya, Ghana, and India. Lancet Glob Heal.

[3] Rosen HE, Lynam PF, Carr C, Reis V, Ricca J, Bazant ES (2015). Direct observation of respectful maternity care in five countries: A cross-sectional study of health facilities in East and Southern Africa. BMC Pregnancy Childbirth..

[4] Asefa A, Bekele D (2015). Status of respectful and non-abusive care during facility-based childbirth in a hospital and health centers in Addis Ababa, Ethiopia. Reprod Health..

[5] Wassihun B, Zeleke S (2018). Compassionate and respectful maternity care during facility based child birth and women’s intent to use maternity service in Bahir Dar, Ethiopia. BMC Pregnancy Childbirth.

[6] Endale Erko M, Abdulahi D, Hiko K, Seid B, Admassu, Compassion MS (2018). Respect and Caring : a Scoping Review of Health Professionals Behavior in Healthcare Delivery in Sub-Saharan. WORLD J Adv Healthc Res.

[7] Munawar A, Hassan ZU, Ayub A, Shaikh BT, Buriro NA, Ahmed F (1970). Women’s perceptions about quality of maternity care at tertiary care hospital Karachi, Pakistan. Pakistan J Public Heal.

[8] Banks KP, Karim AM, Ratcliffe HL, Betemariam W, Langer A (2018). Jeopardizing quality at the frontline of healthcare: Prevalence and risk factors for disrespect and abuse during facility-based childbirth in Ethiopia. Health Policy Plan.

[9] Tunçalp, Were WM, Maclennan C, Oladapo OT, Gülmezoglu AM, Bahl R (2015). Quality of care for pregnant women and newborns - The WHO vision. BJOG An Int J Obstet Gynaecol.

[10] Kruk ME, Kujawski S, Mbaruku G, Ramsey K, Moyo W, Freedman LP (2018). Disrespectful and abusive treatment during facility delivery in Tanzania: A facility and community survey. Health Policy Plan.

[11] Afulani PA, Sayi TS, Montagu D (2018). Predictors of person-centered maternity care: The role of socioeconomic status, empowerment, and facility type. BMC Health Serv Res.

[12] Oladapo OT, Tunçalp, Bonet M, Lawrie TA, Portela A, Downe S (2018). WHO model of intrapartum care for a positive childbirth experience: transforming care of women and babies for improved health and wellbeing. BJOG An Int J Obstet Gynaecol.

[13] Afulani PA, Diamond-Smith N, Golub G, Sudhinaraset M (2017). Development of a tool to measure person-centered maternity care in developing settings: Validation in a rural and urban Kenyan population. Reprod Health.

[14] Afulani PA, Diamond-Smith N, Phillips B, Singhal S, Sudhinaraset M (2018). Validation of the person-centered maternity care scale in India Prof. Suellen Miller. Reprod Health.

[15] Bohren MA, Hunter EC, Munthe-kaas HM, Souza JP, Vogel JP (2014). Facilitators and barriers to facility-based delivery in low- and middle-income countries: a qualitative evidence synthesis. Reprod Health..

[16] Rosen HE, Lynam PF, Carr C, Reis V, Ricca J, Bazant ES (2015). Direct observation of respectful maternity care in five countries: A cross-sectional study of health facilities in East and Southern Africa. BMC Pregnancy Childbirth.

[17] Wassihun B, Deribe L, Worede N, Gultie T (2018). Prevalence of disrespect and abuse of women during child birth and associated factors in Bahir Dar town, Ethiopia. Epidemiol Health.

[18] Sudhinaraset M, Afulani P, Diamond-Smith N, Bhattacharyya S, Donnay F, Montagu D (2017). Advancing a conceptual model to improve maternal health quality: The person-centered care framework for reproductive health equity. Gates Open Res.

[19] Bhattacharya S, Sundari Ravindran TK (2018). Silent voices: Institutional disrespect and abuse during delivery among women of Varanasi district, northern India. BMC Pregnancy Childbirth.

